# Mitigation of Particulate Matter-Induced Inflammation and Vasoactivity in Human Vascular Endothelial Cells by Omega-3 Polyunsaturated Fatty Acids

**DOI:** 10.3390/ijerph15102293

**Published:** 2018-10-19

**Authors:** Jaya Sriram, Olorunfemi Adetona, Tonya Orchard, Chieh-Ming Wu, James Odei

**Affiliations:** 1 Division of Environmental Health Sciences, College of Public Health, The Ohio State University, Columbus, OH 43210, USA; sriram.13@osu.edu (J.S.); wu.1615@osu.edu (C.-M.W.); 2 Human Nutrition Program, Department of Human Sciences, College of Education and Human Ecology, The Ohio State University, Columbus, OH 43210, USA; orchard.6@osu.edu; 3 Division of Biostatistics, College of Public Health, The Ohio State University, Columbus, OH 43210, USA; odei.3@osu.edu

**Keywords:** particulate matter, omega-3, polyunsaturated fatty acid, inflammation, vascular function, mitigation

## Abstract

Airborne particulate matter (PM) exposure remains the leading environmental risk factor for disease globally. Interventions to mitigate the adverse effects of PM are required, since there is no discernible threshold for its effects, and exposure reduction approaches are limited. The mitigation of PM (specifically diesel exhaust particles (DEP))-induced release of pro-inflammatory cytokines interleukin-6 (IL-6) and interleukin-8 (IL-8) and vasoconstrictor endothelin-1 (ET-1) after 24 and 48 h of exposure by pre-treatment with individual pure, combined pure, and an oil formulation of two fish oil omega-3 polyunsaturated fatty acids (ω-3 PUFAs), docosahexaenoic acid (DHA), and eicosapentaenoic acid (EPA) were all tested at an equivalent concentration of 100 µM in vitro in human umbilical vein endothelial cells. The PUFAs and fish oil formulation completely mitigated or diminished the DEP-induced release of IL-6, IL-8, and ET-1 by 14–78%. DHA was more effective in reducing the levels of the DEP-induced release of the cytokines, especially IL-6 after 48 h of DEP exposure in comparison to EPA (*p* < 0.05), whereas EPA seemed to be more potent in reducing ET-1 levels. The potential of fish ω-3 PUFAs to mitigate PM-induced inflammation and vasoactivity was demonstrated by this study.

## 1. Introduction

Exposure to particulate matter-related air pollution is the leading environmental risk factor for disease, which accounted for an estimated 6.8 million deaths and 7.4% of the disability-adjusted life years (DALYs) globally in 2010 [1]. Ambient PM_2.5_ (particulate matter (PM) with aerodynamic diameters of less than 2.5 µm) was also ranked the fifth and sixth risk factor overall for global mortality and DALYs respectively in 2015 [2]. Inhaled PM_2.5_ causes local effects in the respiratory airways [3], and it is capable of inducing systemic effects, apparently due to its ability to translocate into circulation beyond the lungs because of its small size [4]. Exposures to PM_2.5_ in ambient and household air have been associated with mortality and morbidity, mainly due to cardiovascular and respiratory diseases.

Accordingly, the goal of ensuring a low ambient PM concentration is very desirable. However, low- and middle-income countries often lack the control technologies and/or the regulatory regimes to achieve such conditions [5,6]. Although mean annual concentrations at or below the World Health Organization air quality guidelines (10 µg/m^3^) have been achieved in high-income countries of Western Europe and North America [7,8], evidence from recent studies continues to suggest health effects of exposure, even at such low concentrations [2,4]. A threshold exposure for the adverse health effects of PM_2.5_ is also currently indiscernible, especially with respect to the cardiovascular system [4]. Therefore, it is imperative that interventions that mitigate the health effects of PM be developed, in addition to existing exposure reduction efforts [4,9,10,11,12].

Although the understanding of mechanisms underlying the induction of adverse health outcomes by PM remain incomplete, current knowledge indicates that oxidative stress and inflammation are integral in its contribution to various pathologies [10]. Therefore, omega-3 polyunsaturated fatty acids (ω-3 PUFAs), due to their possession of antioxidant and anti-inflammatory properties, could be good candidates for mitigating PM-induced effects [10,13,14]. In addition, ω-3 PUFAs display pleiotropic benefits in the cardiovascular system, including positive effects on vascular function that may counteract other adverse responses mediated by PM [13].

In the current study, we tested and compared the potential of eicosapentaenoic acid (EPA) and docosahexaenoic acid (DHA), the major component ω-3 PUFA of fish oil, to mitigate PM-induced inflammation in vitro in human umbilical vein endothelial cells (HUVECs) across two exposure periods (24 and 48 h). Additionally, we tested the potential of these two ω-3 PUFAs to inhibit the PM-induced release of endothelin-1 (ET-1), a potent vasoconstrictor [15].

## 2. Materials and Methods

### 2.1. Chemicals and Cells

DHA and EPA were purchased from Cayman Chemicals, Ann Arbor, MI, USA. Diesel exhaust particles (DEP, NIST 2995), HUVEC, and endothelial cell growth media (Catalog No. 211-500) were obtained from Sigma-Aldrich, St. Louis, MO, USA. The fatty acid oil formulation was composed of fish oil with a 4:6 ratio of DHA and EPA. The oil formulation is hereafter referred to as OSU45, and was custom made by Marine Ingredients, Mount Bethel, PA, USA. It was generously provided by Dr. Tonya Orchard.

### 2.2. Fatty Acid Oil Saponification

The fatty acid oil formulation was saponified by the method described by Igarashi and Miyazawa (2000) [16]. The formulations (90 mg each) were incubated in a test tube with nitrogen gas for 15 s. Potassium hydroxide (15 mL; 0.3 N) in 90% methanol was then added and the reaction was incubated at 37 °C for 2 h. The cooled reaction mixture was added to 5 mL of 90% methanol and 15 mL of hexane, and shaken vigorously. Non-saponaceous matter was excluded by washing the methanolic aqueous layer twice with 15 mL of hexane. Hydrochloric acid (9 mL; 6 N) was added to the washed methanolic layer. The fatty acids were obtained by extracting twice with 15 mL of hexane and then evaporating the hexane under a nitrogen stream.

### 2.3. Stock Preparation

The DEP stock was made fresh before conducting the experiments, and it was used up within an hour. DEP was weighed and resuspended in appropriate volume of endothelial growth media. The stock was sonicated for 10 min on ice. DEP stock was kept in an ultrasonic bath until usage, to prevent particle aggregation. The OSU fatty acid oil formulation (OSU45) and the pure ω-3 PUFAs (DHA and EPA) were weighed and dissolved in the appropriate amount of endothelial growth media. The stocks were stored at −20 °C until used.

### 2.4. Cell Culture

HUVECs were grown in the endothelial cell growth media at 37 ± 1 °C, 5% CO_2_, 90 ± 2% in a humidity incubator. Exposure experiments were performed between 3–5 cell passages.

### 2.5. Experimental Design

**Figure 1:**
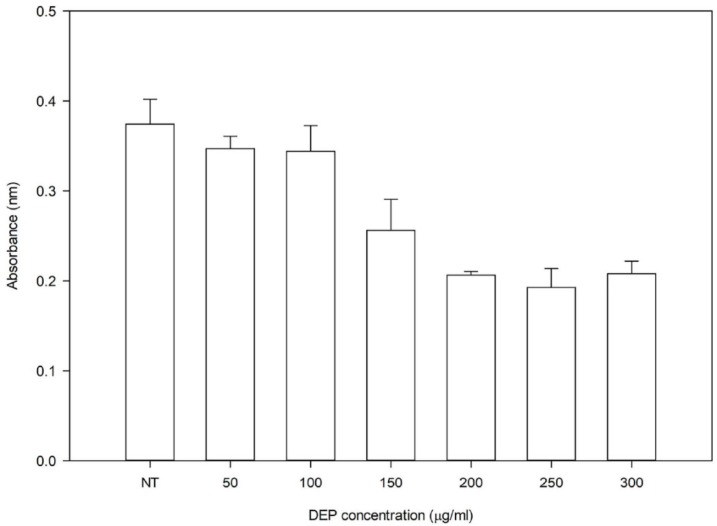
Lactate dehydrogenase assay to determine the diesel exhaust particles (DEP) concentration for experiments. NT is no treatment or DEP concentration at 0 µg/mL.

Cells were exposed to 0–300 µg/mL of DEP to test its effects on cell viability using the lactase dehydrogenase (LDH) assay. There was no difference in cell viability until 100 µg/mL of DEP, with viability decreasing when the concentration was further increased (Figure 1). A final concentration of 100 µg/mL was chosen for exposure experiments, as it was the highest concentration tolerated by cells with no effect on their viability (Figure 1). Pure DHA and EPA were used at a final concentration of 100 µM (50 µM each in the combination), which was similar to the concentrations used in other studies [9,17,18,19,20]. We tested and verified using the LDH assay that DHA and EPA at concentration of 100 µM did not affect cell viability. The fatty acid oil formulation OSU45 was used at a final equivalent concentration of 100 µM, with respect to the most abundant fatty acid components, DHA and EPA (approximately 56%).

The cells were grown in 96-well plates and treated with the fatty acids at 70–80% confluence. After 24 h of fatty acid treatment, cells were exposed to DEP. The culture media was collected after 24 and 48 h of DEP exposure for measuring the levels of endothelin-1 (ET-1), Interleukin-6 (IL-6), and Interleukin-8 (IL-8). Cells not exposed to DEP and the fatty acids (referred to as NT) and cells exposed to only DEP were used as control.

### 2.6. Enzyme-Linked Immunosorbent Assays (ELISA)

The levels of IL-6, IL-8, and ET-1 were determined by ELISA using kits purchased from Thermofisher Scientific, Waltham, MA, USA. The ELISAs were performed according to manufacturer’s instructions.

### 2.7. Statistical Analysis

The mitigation of PM-induced effects by the pure ω-3 PUFAs or their oil formulations were statistically analyzed as follows. The media concentrations of each of the biomarkers (IL-6, IL-8, and ET-1) in each of the ω-3 PUFA treatments (pure and oil formulation) was compared with the concentrations in the controls (DEP only and NT) using one-way analysis of variance, and with the type of treatment as the categorical independent variable. Bonferroni correction was applied for the multiple pairwise comparisons of effects of the ω-PUFA treatments to control for the family-wise error rate of making a type I error.

## 3. Results

### 3.1. Effect of DEP

**Figure 2:**
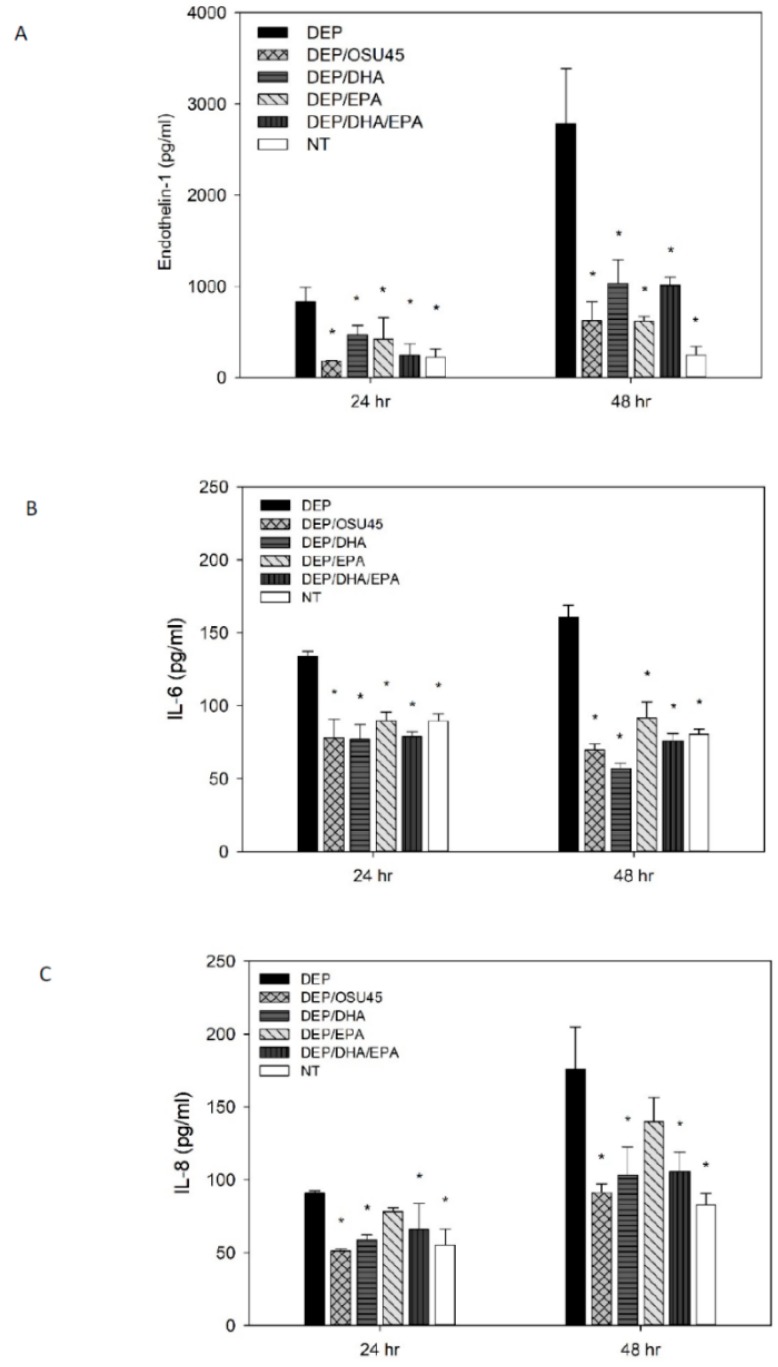
The effect of pure polyunsaturated fatty acids (ω-3 PUFAs), docosahexaenoic acid (DHA), and eicosapentaenoic acid (EPA), their combination (DHA/EPA) or oil formulation containing both (OSU45) at a concentration of 100 µM on DEP-induced release of endothelin-1 (ET-1) (**A**), interleukin-6 (IL-6) (**B**) and interleukin-8 (IL-8) (**C**). NT is the no treatment control without DEP and without ω-3 PUFA treatment. * Treatment is significantly different (*p* < 0.05) from DEP.

The concentration of the vasoconstrictor ET-1 in the harvested media was increased by approximately three-fold after 24 h of exposure to DEP and approximately ten-fold after 48 h of exposure (*p* < 0.05 in pairwise comparison to NT) (Figure 2). A similar trend across the exposure period but more modest increases were observed for IL-6 and IL-8. Their concentrations increased by approximately two-fold after 48 hours of DEP exposure (*p* < 0.05 in pairwise comparison to NT) (Figure 2).

### 3.2. Effect of ω-3-PUFAs

Treatment by pure DHA and EPA, their combination, or the oil formulation containing the two ω-3 PUFAs was effective across the 48-hour exposure period in mitigating DEP-induced release of the pro-inflammatory cytokines and the vasoactive ET-1 (*p* < 0.05 in pairwise comparison to DEP) (Figure 2). All the ω-3 PUFA treatments completely abrogated IL-6 and ET-1 at the two time-points, with biomarker concentrations for ω-3 PUFA treatments being significantly less than (*p* < 0.05) or not different from NT (*p* > 0.05 in pairwise comparison to NT) at the two time points (Table 1). The treatments by the pure fish oil ω-3 PUFAs (EPA and DHA) and their oil formulation reduced DEP-induced release of ET-1 by 44–78%, and IL-6 by 33–64% over the 48-hour period. In addition, all the ω-3 PUFA treatments except EPA completely abrogated IL-8 release (*p* > 0.05 in pairwise comparison to NT) at the two time points (Table 1). A higher degree of mitigation of DEP-induced release of pro-inflammatory cytokines, especially for IL-6 at the 48-hour time-point (*p* < 0.05), was observed with pure DHA treatment compared to pure EHA, while the reverse, although not statistically significant, seemed true for ET-1 (Table 1).

## 4. Discussion

In the current study, pre-treatment with pure ω-3 PUFAs, EPA, and DHA, a combination of them, and an oil formulation containing both were effective at reducing the PM-induced release of pro-inflammatory cytokines and the potent vasoconstrictor ET-1.

Previous studies have demonstrated increased pro-inflammatory and vasoactive effects of PM, with an increasing contact time of up to 64 h in lung epithelial cells, macrophages, and pneumocytes [21,22,23]. Our results demonstrate similar effects in HUVECs, a representative endothelial cell line of the vasculature (Figure 2). The time-dependent effect was particularly strong for ET-1, with a tripling of the PM-induced release at the 48 h time point, compared to levels at the 24 h time point. The corresponding increases for IL-6 (~25%) and IL-8 (~100%) were more modest. Accordingly, we tested whether the mitigative effects of the ω-3 PUFAs persisted beyond the 24 h period up until the 48 h exposure time point.

The pure ω-3 PUFAs (DHA and EPA), their 50:50 (molar ratio) mixture, and fish oil formulation containing a 40:60 (molar ratio) of the fatty acids were effective at eliminating or reducing the tested PM-induced effects across the two-time points. Therefore, the results suggest the persistence of their potencies in the experimental model. The results are supported by those reported in other studies. PM-induced release of IL-6 and another pro-inflammatory cytokine, tumor necrosis-α (TNF-α) in HUVECs was reduced by pre-treatment of the cells with EPA in a recently published study [9]. The relative reduction that was observed only at the highest EPA concentration (30 mg/L) tested in this previous in vitro study is a lot less than what is observed in our study. Although this EPA concentration is equivalent to the 100 mM ω-3 PUFA concentration tested in our study, the difference in the magnitude of the mitigative effects might have been due to the higher PM exposure (100 µg/mL vs. 50 µg/mL) in the current study and the greater degree of responses that it elicited.

Plasma concentrations of pro-inflammatory cytokines (monocyte chemoattractant protein [MCP-1], TNF-α, IL-1β, and IL-6) were also reduced in C57BL/6 wild type mice exposed to PM through intratracheal instillation for six weeks that were either fed a diet supplemented with a mixture of plant and fish oil ω-3 PUFAs for one week prior to exposure, or that were enabled to endogenously produce ω-3 PUFAs by the possession of the Fat-1 transgene [10]. Unlike the observations in the previous in vitro study by Bo et al. (2016), reductions in the mice study were as dramatic as the ones we report. In addition, PM-induced oxidative stress was reduced by the ω-3 PUFAs in the in vivo study.

Results from a minimal number of observational studies of middle-aged and older adults (50–96 years) also suggest the potential of fish oil ω-3 PUFAs to mitigate PM-associated cardiovascular-relevant effects [11,12,24]. Fish oil supplementation for four months in the presence of indoor air PM exposure reduced oxidative stress (plasma lipoperoxidation products) and increased antioxidant activity (Cu/Zn superoxide dismutase enzyme activity and increased plasma concentration of reduced glutathione) [11]. Additionally, a five-month long supplementation with fish oil mitigated PM-associated alteration of cardiac autonomic function (reductions in the time and frequency domain components of heart rate variability) [24]. Although no effect of a four-week supplementation on PM-associated increase in plasma concentrations of pro-inflammatory cytokines was observed in an acute chamber exposure study, the supplementation attenuated acute PM-associated increase in plasma concentration of ET-1 [12].

The mitigative potential of the two primary fish oil ω-3 PUFAs (pure EPA and DHA) against PM-induced responses were examined concurrently in this study, as there is a difference in the capability of making the two primary fish oil ω-3 PUFAs biologically accessible. While DHA is almost exclusively available to humans through the consumption of fish, EPA can be synthesized from the shorter chain plant-based ω-3 PUFAs [25]. Furthermore, there is evidence of their differential effects in the cardiovascular system [25]. However, their differential mitigation of environmentally induced effects have not been investigated. DHA seems to be more potent at mitigating PM-induced release of pro-inflammatory cytokines, IL-6 and IL-8, compared to EPA. This difference is more pronounced at the 48 h time point. Similar results have been reported for the effects of the two fish oil ω-3 PUFAs on lipopolysaccharide-induced inflammation in human THP-1 derived macrophages [18]. On the other hand, EPA seems more potent at mitigating PM-induced release of ET-1 at the 48 h time point. Although these results suggest differential mitigation by fish oil ω-3 PUFAs of the adverse effects of PM in the vasculature, they also demonstrate that the effects are shared and complementary. Consequently, the results indicate that more protection against PM-induced adverse effects in the vasculature would be derived from the consumption of the two fish oil ω-3 PUFAs, rather than only one of them.

The PM exposure concentration of 100 µg/mL (the equivalent unit area concentration in 0.3 cm^2^ 96-well plates of 200 µL experimental media is 66 µg/cm^2^) was comparable to the concentrations used in many in vitro studies of PM-induced inflammation, including the previous study on mitigation by EPA [9,22,26,27]. Nonetheless, the in vitro exposure is unrepresentative of human experience. Extrapolation based on a model-derived estimate suggests that the equivalent air concentration of exposure will be at the high end of the range of human exposure (~100 µg/m^3^), even with the assumption of a safety factor of 10,000 [28]. However, results of the current study still demonstrate that ω-3 PUFAs can mitigate the inflammatory and vasoactive effects of PM at such high levels of exposure.

Although these were not investigated in the current study, potential mechanisms underlying the observed mitigation of inflammatory effects of PM by fish oil ω-3 PUFAs have been elucidated. Reactive oxygen species produced due to PM exposure can cause the entry of fatty acids, primarily arachidonic acid (ARA), which is a ω-6 PUFA, into the eicosanoid synthesis pathway [14]. The availability and subsequent incorporation of ω-3 PUFAs into the cell membrane makes them available to cyclooxygenase-2 and arachidionate-5-lipoxygenase enzymes in competition with ARA as precursors for eicosanoid synthesis. The prostaglandins and leukotrienes synthesized from fish oil ω-3 PUFAs are less potent mediators of inflammation, compared with those from ARA [14]. Additionally, ω-3 PUFAs inhibit the activation of nuclear factor kappa B (NF-ĸB) [14], which is the transcription factor primarily responsible for the inflammatory effects of PM [29]. Lastly, more potent mediators of the resolution of inflammation are produced from ω-3 PUFAs, compared to ARA through cyclooxygenase-2 and lipoxygenase enzymatic activities [14]. These mechanisms in conjunction with PM exposure and the potential of other ω-3 PUFAs from alternative (plant) sources will be investigated in future studies.

## 5. Conclusions

The mitigation of PM-mediated inflammatory and vasoconstriction-related effects in HUVECs by ω-3 PUFAs in this study indicates their potential to be used in conjunction with exposure-reduction approaches for controlling PM-associated adverse health effects.

## Figures and Tables

**Table 1:** Comparisons between ω-3 PUFA treatments and the no treatment control (NT) using the Bonferroni correction for multiple comparisons.

Comparisons	24-Hours		48-Hours	
	*t*-Value	*p*-Value	*t*-Value	*p*-Value
	***ET-1***			
DHA vs. NT	2.20	0.24	3.33	0.30
EPA vs. NT	1.75	0.53	1.58	0.71
OSU45 vs. NT	−0.32	1.00	1.62	0.66
DHA vs. EPA	0.45	1.00	1.75	0.53
DHA vs. OSU45	2.52	0.13	1.71	0.56
EPA vs. OSU45	2.07	0.30	−0.04	1.00
	***IL-6***			
DHA vs. NT	−1.98	0.35	−4.33	**<0.01**
EPA vs. NT	0.05	1.00	2.08	0.30
OSU45 vs. NT	−1.82	0.47	−1.96	0.37
DHA vs. EPA	−2.03	0.33	−6.41	**<0.01**
DHA vs. OSU45	−0.16	1.00	−2.37	0.18
EPA vs. OSU45	1.87	0.43	4.04	**<0.01**
	***IL-8***			
DHA vs. NT	0.53	1.00	1.46	0.83
EPA vs. NT	3.19	**0.04**	2.08	**<0.01**
OSU45 vs. NT	−0.55	1.00	0.60	1.00
DHA vs. EPA	−2.66	0.10	−2.61	0.11
DHA vs. OSU45	1.08	1.00	−0.86	1.00
EPA vs. OSU45	3.74	0.01	−3.47	**0.02**

Definitions: DHA—docosahexaenoic acid; EPA—eicosapentaenoic acid; OSU45—fish oil formulation containing DHA and EPA; NT—no treatment control without DEP and without ω-3 PUFA treatment; ET-1—endothelin-1; IL-6—interleukin-6; IL-8—interleukin-8. *p*-values for comparisons that are significant (*p* < 0.05) are in bold font.
